# Comparison of hydrability, antioxidants, microstructure, and sensory quality of barley grass powder using ultra‐micro‐crushing combined with hot air and freeze drying

**DOI:** 10.1002/fsn3.2138

**Published:** 2021-02-05

**Authors:** Wei Zhou, Xiaohuang Cao, Md. Nahidul Islam, Huiting Zheng, Jihua Li, Fei Liu, Yupo Cao, Yaping Dai

**Affiliations:** ^1^ Key Laboratory of Tropical Crop Products Processing of Ministry of Agriculture and Rural Affairs Agricultural Products Processing Research Institute Chinese Academy of Tropical Agricultural Sciences Zhanjiang China; ^2^ Hainan Key Laboratory of Storage and Processing of Fruits and Vegetables Zhanjiang China; ^3^ College of Chemistry and Food Yulin Normal University Yulin China; ^4^ Department of Agro‐Processing Bangabandhu Sheikh Mujibur Rahman Agricultural University Ghazipur 1706 Bangladesh; ^5^ School of Chemistry and Chemical Engineering Lingnan Normal University Zhanjiang China

**Keywords:** barley grass, drying methods, physicochemical properties, sensory quality, ultra‐micro‐crushing

## Abstract

To explore the physicochemical characters of barley grass, ultra‐micro‐crushing (UMC) technology combined with air drying or freeze drying was carried out. After barley grass was air‐dried at 70°C or freeze‐dried at 15°C, it was grinded for 30, 60, 90, and 120 min using UMC, respectively. After combined processing, moisture content, particle size, odor, color, microstructure, water and oil‐holding capacity, the content of flavonoid and chlorophyll, water activity, and sensory qualities were determined. The particle size of barley grass powder decreased, and lightness value was increased; water and oil‐holding capacity decreased significantly (*p ≤ *.05), whereas swelling and dissolving capacity increased in the processed grass powder. On the other hand, the total flavonoid content increased significantly (*p* ≤ .05). Barley grass odor features sulfide aroma, and its microstructure demonstrates lamellar morphology with some fewer fragmented pieces. The results suggested combined UMC at 90–120 min will be suitable for processing barley grass powder.

## Graphical abstract

UMC technology combined with different drying methods has a great progress in the future processing of barley grass powder.

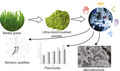

## 1 INTRODUCTION

Barley grass (*Hordeum vulgare* L.) is mainly cultivated in the temperate and subtropical regions of the world; especially, it is commonly cultivated in China. Barley grass powder refers to products of young barley grass processed by picking, cleaning, cutting, drying, and grinding, which produces dark green powder. Zeng et al. (2018) found that barley grass powder is rich in chlorophyll, protein, vitamins, minerals, and other nutrients. In particular, it is high in antioxidants, chlorophyll, flavonoids, and dietary fiber (Cao et al., 2017, 2019; Mujoriya & Bodla, 2011). Barley grass powder is helpful in controlling some diseases, such as diabetes and rheumatic disease (Rana et al., 2011). Industries are producing barley grass powder in China; thus, processing of the barley grass powder is a hot topic of research in the recent years (Akbas et al., 2017; Cao et al., 2018a). At present, the major problem of the products is lower water solubility and reduced content of functional compounds (Cao et al., 2018d; Chouhan & Mogra, 2014). Thus, the processing of barley grass powder still needs development (Cao et al., 2018c). Therefore, to enhance the quality of barley grass powder, researching advance processing and the solid understanding of the process are of demand.

With the development of processing technology, the novel ultra‐micro‐crushing (UMC) technology is becoming popular, which increases surface area, enhances the surface activity, and possesses materialization characteristics (YaSha, 2004). Scientists are introducing UMC technology to pharmaceuticals and food materials (Cao et al., 2018b; Jianrong et al., 2007; Yanli, 2008). However, the comparison of UMC combined with freeze or air drying on the quality of barley grass powder has not been investigated.

In this paper, the effect of UMC treatment in two drying methods on the physiochemical properties of barley grass powder was studied. Powder particle size, chromatic aberration, and chlorophyll and total flavonoid were examined, and the change in the powder properties in the process of UMC of barley grass powder was briefly investigated.

## 2 MATERIALS AND METHODS

### 2.1 Materials and methods

Barley grasses were cultivated at seven months in Jiangsu area and harvested by Jiangsu Dingneng Co., Ltd. Barley grasses were then transported to the laboratory by ice‐pack within 48 hr, and stored in the refrigerator at 5°C.

#### 2.1.1 Processing barley grass

**Figure 1:**
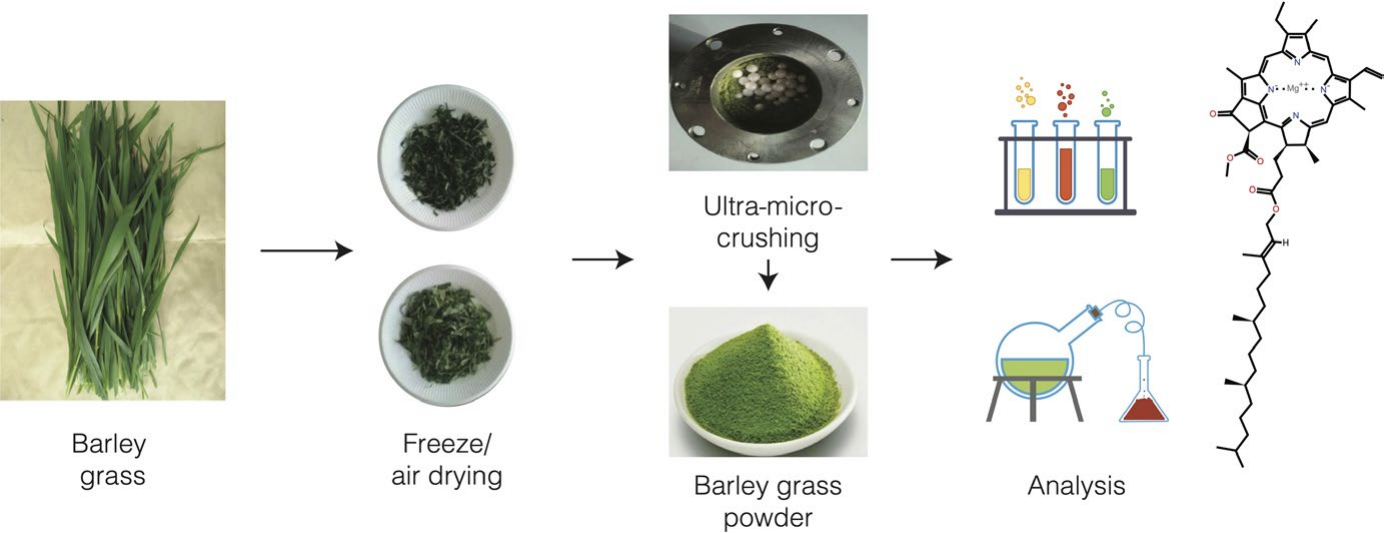
Flow diagram of barley grass processing

The flow diagram of the barley grass processing has shown in Figure 1. Ten kg barley grasses was taken and cleaned with purified water. After cleaning, these grasses were cut into 2‐cm pieces and then were placed onto two plastic trays (1 m × 1 m) with 2 cm thickness. One of the grass‐filled plastic tray was put into drying oven (FP115 thermostatic drying box; German Binder Company), and these grasses were air‐dried at 70°C until it contains 5% moisture content; the other filled plastic tray was put into freeze drying chamber, and the grasses were freeze‐dried at 15°C and at 50 Pa for 8 hr. After air drying/freezing drying (AD/FD), dried grasses were then pulverized using high‐speed crusher at 3,000 rpm for 3 min. Pulverized powders were then sieved by 500 mesh, the rest (over 500 mesh) was again pulverized at 3,000 rpm for 3 min; then, the rest over 500 mesh was repeated for pulverizing and sieving until all passed 500 mesh barley grass powder. AD/FD 500 mesh samples were placed in the ball mill (F‐P400E miniature all‐round planetary ball mill; Hunan Fukas Test Instrument Co., Ltd.) as described by Cao et al. (2018b). The ball grinding time was set for 30, 60, 90, and 120 min, to get eight kinds of ultra‐micro‐crushed powder marked as AU3, AU6, AU9, and AU12 and FU3, FU6, FU9, and FU12, respectively. Processed powders were vacuum‐packed in 100 g unit using a foil bag and stored at 5°C for further examination.

### 2.2 Particle size determination

The particle size measurement was carried out using a laser particle size analyzer (Mastersizer 2000) purchased from Malvern, UK. 0.05 g barley grass powder was added in 10 mLdistilled water, after homogeneous mixing, 1 mL amount was taken in the measuring container, and the particle size was measured as described by Shu et al. (2007).

### 2.3 Determination of color

The color measurement system was calibrated against white and blackboards before measurement. The sample color was measured as described by Cao, Islam, Xu, et al. (2020). The values of L^*^, a^*^, and b^*^ were noted down. L^*^ value represents the brightness of the object, the higher the score, the whiter the sample is, a^*^ value represents redness to greenness of the object, positive value represents redness, while negative value represents greenness, b^*^ value represents yellowness to blueness, positive value represents yellowness, and negative value represents blueness (Islam et al., 2014). Each sample was measured three times.

### 2.4 Determination of chlorophyll content

Accurately weighted 0.50 g of barley grass powder was taken in a 250‐mL triangular bottle, and 100 mL extractor (1:1/V:V mixture with ethanol and acetone) was added to it. Triangular bottle was sealed with sealed film and kept for 5 hr at 25°C for extraction. The extract was then centrifuged at 2191 g for 15 min; after centrifugation, the aliquot was poured out for testing. The zero ingest point was adjusted with blank solution, and the absorbent value was recorded at 645 and 663 nm as described by Nagata and Yamashita (1992). Chlorophyll a, b content and total chlorophyll content were calculated using Equations ((1), (2), (3)).

(1) $$\omega_{1}=\frac{(12.72\times A_{1}‐2.59\times A_{2})\times v}{1000\times m}$$

(2) $$\omega_{2}=\frac{(22.88\times A_{2}‐4.67\times A_{1})\times v}{1000\times m}$$

(3) $$\omega_{3}=\frac{(8.05\times A_{1}+20.29\times A_{2})\times v}{1000\times m}$$

where $\omega_{1}$ is the chlorophyll a content (mg/g), $\omega_{2}$ is the chlorophyll *b* content (mg/g), and $\omega_{3}$ is the total chlorophyll content (mg/g), *v* is total volume of extract, A_1_ is absorbance value in 663 nm, and A_2_ is absorbance value in 645 nm.

### 2.5 Measurement of water‐holding capacity

Powder sample (0.50 g) was dissolved into 20 mL distilled water and oscillated with ultrasound for 30 min to obtain homogeneity. After homogeneous turbulence, mixture was transferred to the 50‐mL centrifuge tube and centrifuged at 2191 g for 20 min. After centrifugation, supernatant was poured out and sediment was taken for vacuum drying and final weighing mass of residue. Water‐holding capacity was calculated according to Equation (4).

(4) $$\text{WHC}=\frac{m_{2}‐m_{1}}{m_{1}}$$

where WHC presents water‐holding capacity, g/g; *m*_2_ presents mass of residue after dissolution, separation, and vacuum drying, g; *m*_1_ presents mass of samples, g.

### 2.6 Measurement of oil‐holding capacity

Barley powder (1.5 g) and 12 mL peanut oil were taken into 50‐mL centrifuge tube and ultrasonically oscillated for 30 min for adequate oil absorption. Then, the mixture was separated by centrifugation at 2191 g for 20 min, and supernatant was poured out and the residue was vacuum‐dried for weighting mass of remnants. Oil‐holding capacity was calculated according to Equation (5).

(5) $$\text{OHC}=\frac{m_{2}‐m_{1}}{m_{1}}$$

where OHC presents oil‐holding capacity, g/g; *m*_2_ presents mass of residue, g; *m*_1_ presents mass of dried samples, g.

### 2.7 Measurement of water swelling capacity

Water swelling capacity was determined according to the method described by Chantaro et al. (2008). Accurately weighted 0.50 g barley grass powder was taken in the glass test tube. Initial volume of the sample was recorded. 10 mL distilled water was added to it and oscillated until evenly dispersed and kept at 25°C in a water bath for 24 hr. The full expansion volume was recorded again. Water swelling capacity was calculated using Equation (6):

(6) $$\text{SC}=\frac{V_{1}‐V_{2}}{m}$$

where SC is the water swelling capacity, *V*_1_ is sample initial volume (mL), *V*_2_ is the swelling sample volume (mL), and m is the sample mass (g).

### 2.8 Measurement of dissolving capacity

Accurately weighted 0.20 g sample was put into 5‐mL tube, and distilled water was added at mass ratio of 0.02:1 (samples: water). The mixture was then placed in a water bath oscillation at 80°C for 30 min and then centrifuged at 2191 g (Sigma 3–30 K low‐temperature high‐speed centrifuges: Sigma) for 20 min. The aliquot was then removed and dried at 105°C until constant weight. Dissolving capacity was then calculated using Equation (7) as shown by Cai et al. (2018)

(7) $$\text{DC}=M_{2}/M_{1}$$

where DC is the dissolving capacity, *M*_1_ is the weight of sample (g), and *M*_2_ is the weight of soluble substance after drying (g).

### 2.9 Determination of total flavonoids

Total flavonoid content was determined by the earlier method (Peñarrieta et al., 2007). The absorbent was measured at wavelength of 510 nm with standard liquid using a spectrophotometer (752N UV‐visible spectrophotometer Shanghai Precision Scientific Instrument meter Co., Ltd) according to Huang et al. (2014). With the corresponding content of the absorbent value, standard curve was drawn at 0, ,0.2, 0.4, 0.6, 0.8 and 1.0 mg/mL, respectively. 1.0 g sample was extracted with 50 mL 80% acetone for 8 hr. 5 mL of the extract (supernatant) was put into a 25‐mL colorimetric tube, 5.0 mL ethanol solution and 1 mL sodium nitrite solution were added and shook well, and rest for 6 min. Then, 1.5 mL aluminum nitrate solution was added to it and shook well, and kept at rest for 6 min, and 4 mL sodium hydroxide solution was added, and then scaled it with water and shook for 15 min. Chromogenic brass solution (1 mL) was measured at 510 nm, and absorbent value was recorded. According to standard curves, corresponding content of flavonoids was calculated. Meanwhile, a blank test was carried out with the corresponding sample fluid without the presence of aluminum nitrate solution.

### 2.10 Determination of odor

Odor was determined by an electronic nose (PEN 3, AIRSENSE) consisting of an array of gas‐sensitive sensors, signal preprocessing and pattern recognition as shown by Cao, Islam, Xu, et al. (2020). The e‐nose has 10 metal oxide semiconductor‐type chemical sensing elements that are sensitive to different types of volatiles: benzene; nitrogen oxides; ammonia; hydrogen compounds; short‐chain alkanes; methyl group; sulfide; alcohol, aldehydes, and ketones; organic sulfides; and long‐chain alkanes. An odor is presented as signals (mV value) by a sensor converting chemical content inputs into electrical signals through signal preprocessing and pattern recognition. In the presence of high content of aroma, the corresponding signal value is high. At first, the barley grass powder was put into a glass tube (about 1.5 mL), and then one of the preheated (30 min) needle of the e‐nose instrument was inserted into the glass tube containing the sample. The other needle was also inserted into the glass tube to balance the air pressure. Before each measurement, the electronic sensor was cleaned for 90 s and the measurements were done for 60 s. Gas injection of samples was maintained at 6 mL/s, each sample was arrayed three times at 25°C.

### 2.11 Measurement of water activity

Water activity (*a_w_*) was measured with a moisture activity meter (FA‐ST/lab; GBX Instrumentation Scientifique) as described by Karásková et al. (2011). 0.50 g of barley grass powder was placed on a moisture activity meter and measured at 15, 20, 25, and 30°C, respectively. Each sample was measured thrice, and the mean values and standard deviations were calculated.

### 2.12 Microstructure

The microstructure of UMC barley grass powder was captured using a scanning electron microscope (SEM) (model SU151, Hitachi High‐Tech) at 10.0 kV accelerating voltage with the following method described by Hirano et al. (1981).

### 2.13 Sensory evaluation

To obtain a comprehensive evaluation of barley grass powder, a group of twenty panelists were selected (ten men, ten women, about 20–40 years old). The sensory evaluation was carried out according to the requirements of the sensory evaluation as described by Cao, Islam, Duan, et al. (2020). Scores on color, smell, tastes, and appearance were collected and calculated in integer for analysis. A 5‐point scale was used for scoring: 4–5, excellent; 3–4, good; 2–3, acceptable; 1–2, fair; and 0–1, unacceptable. The evaluation was performed at sensory evaluation laboratory.

### 2.14 Statistical analysis

Analysis of variance (ANOVA) was applied on the data, and multiple comparisons were carried out using Duncan's multiple range test with the SPSS software (SPSS 20.0, IBM). All diagrams were drawn using the Origin 8.0 software (Origin Lab Corporation, Roundhouse Plaza). All measurements were carried out in triplicate; values were presented as mean values with standard deviations.

## 3 RESULTS AND DISCUSSION

### 3.1 Particle size

Particle size is an essential index regarding physicochemical properties of powder. Table 1 shows the particle sizes affected by different ultra‐micro‐pulverization. With the increasing time of ball grinding, powder size in the ultra‐micro‐AD500 and ultra‐micro‐FD500 samples reduced significantly (*p ≤* .05). More than five times of reduced particle size was obtained by ultra‐micro‐processing. This result indicates that UMC is applicable in the processing of dried barley grass. No significant (*p* ≤ .05) changes in particle size were achieved between 90 and 120 min of UMC, which suggests that at over 90 min, particle size reduction with UMC became hard. Thus, processing time in UMC is recommended to be 90–120 min in barley grass powder. Through UMC, powder in air‐dried grasses was found finer than in freeze drying. The reason might be that crisp in cellulose bundles was dried more by air‐dried grasses than by freeze‐dried grasses, whereas in 30, 60 min, comparison of particle size was inverse. This inverse means freeze‐dried grasses are shattered faster than hot‐aired grasses. This reason might be ascribed to multi‐void structure formed from freeze‐dried grasses, this structure being grind faster easily. From all above discovery, higher crisp of cellulose bundles in air‐dried grasses was speculated and mass of multi‐void structure was inferred in two processing.

**TABLE 1:** Particle size of barley grass powder at different ultra‐micro‐crushing times

Milling time (min)	Particle size of barley grass powder (μm)				
	0	30	60	90	120
AD500 mesh	25.13 ± 0.10	5.04 ± 0.01^A,a^	3.50 ± 0.06^A,b^	3.24 ± 0.01^A,c^	3.18 ± 0.23^A,c^
FD500 mesh	26.62 ± 0.05	3.68 ± 0.17^B,a^	3.44 ± 0.23^B,b^	3.41 ± 0.01^B,c^	3.30 ± 0.15^B,c^

Note

Different uppercase means different in columns; different lowercase means different in rows.

### 3.2 Color changes

As it can be seen from the Table 2, that UMC affected the color values of barley grass powder significantly (*p* ≤ .05). Regardless of the drying method, the L*‐value of the UMC samples increased significantly (*p* ≤ .05); however, sample treated over 90 min in UMC led nonsignificant increase in L* value. This behavior is consistent with the earlier references (Cao, Islam, Xu, et al., 2020; Wang et al., 2016). 500 mesh powder of air‐dried samples was similar to 500 mesh powder of freeze‐dried samples with respect to L* value change. The a* value showed a trend of declining between 30 and 120 min, whereas the b* value showed the same downward trend, but not significant (*p *≤ .05). Similar behavior was observed in the earlier work (Cao et al., 2019). In comparison of both dried samples, changes in b* values followed the similar pattern like a* values. The reason is ascribed to compact surface (from powder density) that enhanced the reflection of light through UMC barley grass. In comparison with non‐ultra‐micro‐mashing samples, ultra‐micro‐mashing resulted in dark green because of deceasing b* values. This result is different to the earlier research in processing barley grass powder (Cao, Islam, Xu, et al., 2020; Cao et al., 2018b). The reason might be due to the higher grinding time than current study, longer time induced browning (Cao, Islam, Xu, et al., 2020; Cao et al., 2018b). Air‐dried/freeze‐dried combined UMC resulted the same behavior in color change.

**TABLE 2:** Effect of ultra‐micro‐crushing combined with air/freeze drying on color of barley grass powder at different times

Code samples	Milling time (min)	Changes of color values of barley grass powder		
		L*	a*	b*
Control AU0	0	67.32 ± 0.48^C^	−11.05 ± 0.76^A^	33.73 ± 0.18^A^
AU3	30	71.49 ± 0.10^B^	−13.22 ± 0.10^B^	24.40 ± 0.12^B^
AU6	60	72.13 ± 0.27^A^	−13.45 ± 0.36^B^	24.22 ± 0.35^B^
AU9	90	72.20 ± 0.23^A^	−13.73 ± 0.32^B^	24.19 ± 0.17^B^
AU12	120	72.29 ± 0.14^A^	−13.70 ± 0.20^B^	24.08 ± 0.25^B^
Control FU0	0	70.16 ± 0.21^d^	−9.20 ± 0.36^a^	20.85 ± 0.33^a^
FU3	30	71.58 ± 0.20^b^	−10.82 ± 0.05^b^	22.50 ± 0.12^a^
FU6	60	71.70 ± 0.15^c^	−10.90 ± 0.56^b^	22.49 ± 0.70^a^
FU9	90	72.05 ± 0.19^a^	−11.04 ± 0.09^b^	21.51 ± 0.07^a^
FU12	120	72.25 ± 0.16^a^	−11.10 ± 0.15^b^	21.07 ± 0.54^a^

Note

Different uppercase in AU column means significantly differences (*p* ≤ .05); different lowercase in FU column means significant difference (*p* ≤ .05); AU0, AU3, AU6, AU9, and AU12 is control case, ADUMC at 30, 60, 90, 120 min; FU0, FU3, FU6, FU9, FU12 is control case, FDUMC samples at 30, 60, 90, 120 min.

### 3.3 Changes in total chlorophyll

**Figure 2:**
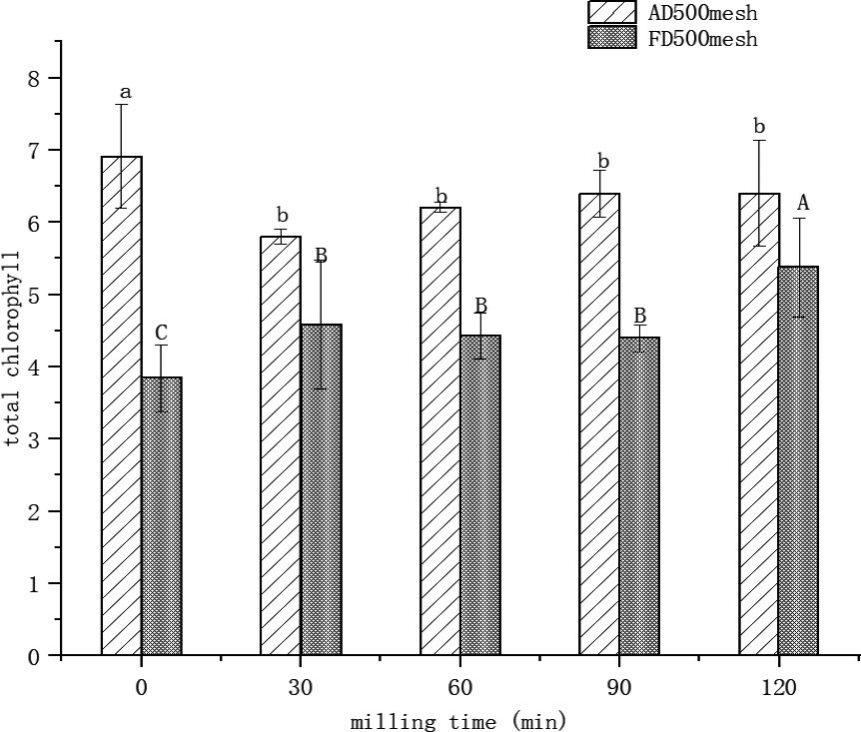
Effects of air drying/freeze drying combined ultra‐micro‐crushing on total chlorophyll in barley grass powder at different times. *Note:* Different letter means significant differences (*p ≤ *.05); AD500 mesh means air‐dried 500 mesh powder; FD500 mesh means freeze‐dried 500 mesh powder

As a natural pigment and food constituents, chlorophyll content is an important index in barley grass powder. Figure 2 shows the effect of different UMC on the total chlorophyll content of barley grass powder. It can be seen in Figure 2 that changes in the total chlorophyll content of AD500 mesh barley grass powder were not obvious. The reason could be due to that ultra‐micro‐crushing (30–120 min) has fewer damages to chlorophyll substance in present study. This result was similar to the earlier research (Havlíková et al., 2014). This reason might be ascribed to different processing, which leads different chlorophyll contents.

With the increase in UMC time, the total chlorophyll content of AD500 shows the trend of sudden drop and then rise, while FD500 mesh samples showed a trend of continuous rise. However, the total chlorophyll content of FD500 mesh was lower than that of the AD500 mesh powder. In FD500 mesh powder, the total chlorophyll content increased significantly (*p *≤ .05) with the increasing time of UMC. In all, chlorophyll content of UMC powder was significantly (*p *≤ .05) lower from non‐UMC powder; surprisingly freeze drying combined UMC (120 min) showed significantly higher chlorophyll content, which decreased in UMC at 30, 60, and 90 min. This behavior meant UMC‐degraded chlorophyll but freeze drying preserved high content of chlorophyll. This phenomenon is ascribed to a large amount of bound chlorophyll existed in FD grasses, which was fully released by UMC. It was found when UMC was compared with the same UMC time, the total chlorophyll content of air‐dried sample was higher than that of freeze‐dried barley grass powder. It is ascribed to obvious damage to barley grass structure from air drying; this causes results in high efficiency in the extraction of chlorophyll (Cao et al., 2018a; Havlíková et al., 2014).

### 3.4 Physical properties

Listed index is important to detect the performance of samples, which can provide a better profile in food powder research. Table 3 presents the different effect of UMC combined with two drying methods on the properties of barley grass powder in terms of water‐holding capacity, oil‐holding capacity, swelling capacity and water solubility. It can be seen from Table 3 that water‐holding capacity and oil‐holding capacity of AD500 mesh and FD500 mesh barley grass powder were reduced significantly (*p *≤ .05), whereas opposite trend was observed in swelling capacity and dissolving capacity. This may be due to UMC degrades the molecular mass of the barley grass, which increase soluble fiber content, and high content of soluble fiber leads to high swelling capacity and dissolving capacity (Cao et al., 2018c, 2018d). However, the fiber degradation in the barley grass destructed numerous fissures and porous structure, and the less fissures and porous structures result in low value of water‐holding capacity and oil‐holding capacity. Other studies found that apple dietary fiber after UMC also appeared a decline in dissolving capacity (Alsuhaibani, 2015; Cao et al., 2018b, 2019). The reason might be different physicochemical properties of barley grass and apple fiber. With the increase in UMC time, dissolving values increased, while that of freeze drying powder was changed nonsignificantly. It might be that high flexibility of cellulose in freeze‐dried samples possesses higher resistance in cellulose destruction with ball grinding (Cao et al., 2018b).

**TABLE 3:** Effects of different ultra‐micro‐crushing time on basic physical properties of barley grass powder

Processing	Code samples	Milling time (min)	Physical properties of barley grass powder			
			Water‐holding capacity (g/g)	Oil‐holding capacity (g/g)	Swelling capacity (ml/g)	Dissolving capacity (%)
ADUMC	AU0	0	6.55 ± 0.14^A^	3.65 ± 0.20^A^	5.08 ± 0.68^B^	20.18 ± 0.54^A^
	AU3	30	3.75 ± 0.21^B^	2.81 ± 0.20^B^	6.58 ± 0.15^A^	30.00 ± 0.10^BC^
	AU6	60	3.45 ± 0.69^BC^	2.75 ± 0.39^B^	6.51 ± 0.12^A^	33.60 ± 0.24^AB^
	AU9	90	3.15 ± 0.94^BC^	2.47 ± 0.53^C^	6.27 ± 0.31^A^	36.70 ± 0.13^BC^
	AU12	120	2.84 ± 0.66^C^	2.40 ± 0.42^C^	6.19 ± 0.20^A^	33.30 ± 0.13^C^
FDUMC	FU0	0	6.46 ± 0.22^a^	3.64 ± 0.16^a^	5.67 ± 0.05^b^	25.30 ± 0.14^a^
	FU3	30	3.21 ± 0.08^b^	2.91 ± 0.33^b^	6.36 ± 0.18^a^	36.71 ± 0.17^a^
	FU6	60	3.12 ± 0.19^b^	2.73 ± 0.29^c^	6.28 ± 0.13^a^	38.35 ± 0.12^a^
	FU9	90	2.85 ± 0.20^b^	2.85 ± 0.19^b^	6.28 ± 0.15^a^	34.31 ± 0.19^a^
	FU12	120	2.86 ± 0.46^b^	2.75 ± 0.41^c^	6.24 ± 0.30^a^	35.00 ± 0.25^a^

Note

Different uppercase means significantly differences (*p ≤ *.05) in ADUMC column; Different lowercase means significant difference (*p ≤ *.05) in FDUMC column; AU0, AU3, AU6, AU9, AU12 is control case, ADUMC at 30, 60, 90, 120 min; FU0, FU3, FU6, FU9, FU12 is control case, FDUMC samples at 30, 60, 90, 120 min.

**TABLE 4:** Sensory acceptability of barley grass powder processed by hot air drying/freeze dying combined ultra‐micro‐pulverization at different times

Sample	Milling time (min)	Sensor acceptability of barely grass powder				
		Color	Smell	Taste	Appearance	Average
AD500 mesh	0	2.14	2.94	1.12	2.02	2.05
	30	1.92	1.96	2.00	1.96	1.95
	60	3.04	2.04	2.08	3.02	2.54
	90	3.06	2.06	2.12	3.12	2.59
	120	3.12	2.14	3.08	3.88	3.05
FD500 mesh	0	1.06	2.08	1.06	1.02	1.31
	30	1.82	1.04	2.10	1.08	1.51
	60	1.98	3.08	1.94	2.04	2.26
	90	3.10	2.12	2.10	3.12	2.61
	120	3.16	2.96	3.08	2.94	3.03

Note

4–5, excellent; 3–4, good; 2–3, acceptable; 1–2, fair; 0–1, unacceptable.

### 3.5 Evaluation of total flavonoid content

**Figure 3:**
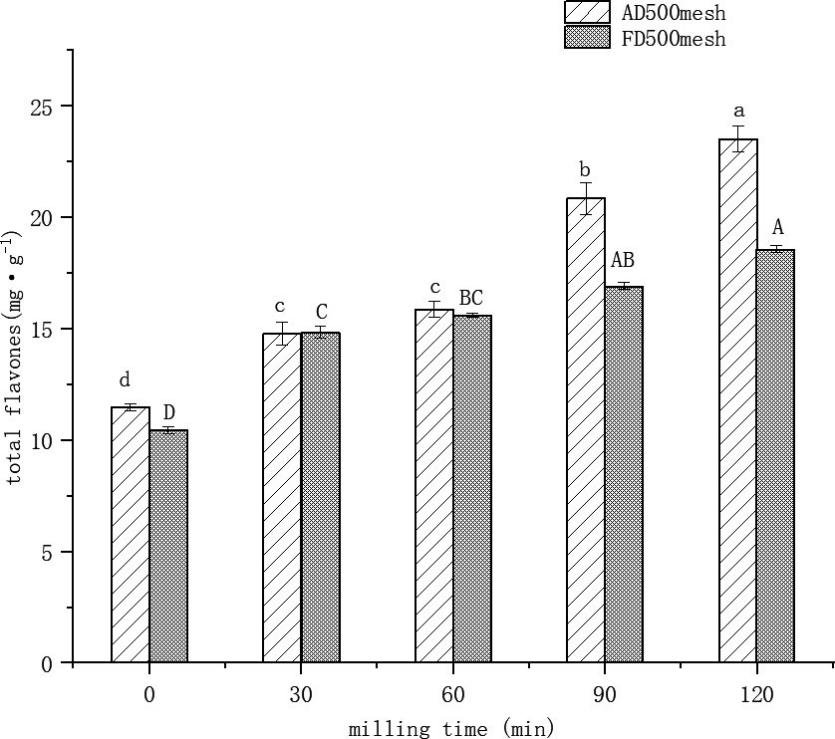
Effects of hot air drying/freeze drying combined ultra‐micro‐crushing on total flavonoids in barley grass powder at different processing times. Note: Different letter means significant difference (*p* < .05); AD500 mesh means air‐dried 500 mesh powder; FD500 mesh means freeze‐dried 500 mesh powder

Flavonoid content is the important element of barley grass powder as a food product. Figure 3 shows the effect of different UMC combined with drying on the total flavonoids content of barley grass powder. For both drying, UMC‐processed barley grass total flavonoid content has significantly increased (*p *≤ .05). This behavior might be due to UMC processing decreased particle size and increases fissures resulting in the release of large free volume of the particles (Kamiyama & Shibamoto, 2012). Interestingly, total flavonoid content in AD500 mesh UMC powder was higher than FD500 mesh UMC‐processed barley grass powder. The behavior is due to increase in brittleness of air‐dried barley grass, which generated more fissures facilitating flavonoids released after UMC treatment processing (Frost et al., 1975).

Additionally with the increasing time of UMC, the total flavonoid content of UMC‐processed barley grass powder also increased significantly (*p* ≤ .05) and total flavonoid content reached the max value of about 23 mg/g and 18 mg/g corresponding to UMC combined with air drying and UMC combined with freeze drying, respectively. Total flavonoid content of this study was lower than that in earlier research (Cao et al., 2018c). This reason might be difference in flavonoids of material along with different growth stages.

### 3.6 Odor assessment

**Figure 4:**
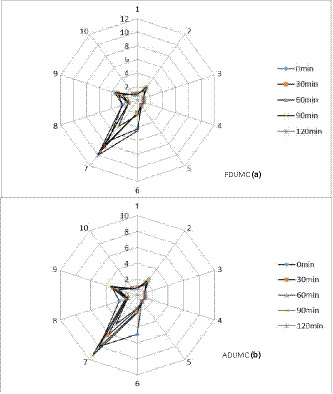
Effects of hot air drying/freeze dying combined ultra‐micro‐pulverization on odor of barley grass powder at different times. *Note:* 1–10 sensor sensitive to these substances. 1, aromatics, benzene; 2, nitrogen oxides; 3, aromatic, ammonia; 4, hydrogen compounds; 5, short‐chain alkanes; 6, methyl group; 7, sulfide; 8, alcohol, aldehydes, and ketones; 9, aromatic components and organic sulfides; 10, long‐chain alkanes. ADUMC means air‐dried ultra‐micro‐crushing samples; FDUMC means freeze‐dried ultra‐micro‐crushing samples

Figure 4 shows the effect of different UMC combined with air/freeze drying on the odor of barley grass powder. Distribution of the main component of barley grass powder odor was depicted at different UMC times. After dried barley grass was ultra‐micro‐crushed, odor distribution demonstrated 9 (long‐chain alkanes) and 7 (sulfide). It is consisted with odor of dehydrated vegetables, which represents elements of vegetables (Cao et al., 2018c). In FDUMC with the increasing of processing time, the signal value of odor showed three behaviors: rise, drop, and then increase.

With UMC time of 90 min, odor distribution area was the smallest, UMC time of 120 min resulted in the largest area of odor distribution. This reason might be 120‐min UMC released mass of methyl, which increased odor area. In air‐dried combined UMC, the odor distribution trend was lower at the beginning and then increased and later decreased. The reason might be due to release rate of aromatic and evaporation rate of flavoring substances. For UMC barley grass of 120 min, odor distribution area was the smallest. It is possible that the long ball grinding time on the air‐dried 500 mesh powder might result in the loss of aroma ingredients, while the FD500 mesh barley grass can retain the aroma as much as possible (Coumans et al., 1994). This result implied UMC combined with freeze drying was superior to UMC combined with air drying in containing odor.

### 3.7 Changes in water activity

**Figure 5:**
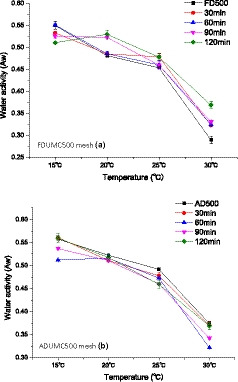
Water activity curves of hot air drying/freeze dying combined ultra‐micro‐crushing in barley grass powder at different temperatures. *Note:* ADUMC means air‐dried ultra‐micro‐crushing samples; FDUMC means freeze‐dried ultra‐micro‐crushing samples

Figure 5 shows the effect of different UMC combined with air/freeze drying on the moisture activity of barley grass. As it can be seen from Figure 5, water activity was different with grinding schemes while the *a_w_* value decreased slowly and then rose fast with temperature rising. Water activity of both samples showed a decreasing trend with increasing temperature. This reason is mainly ascribed to storage temperature (Magan & Lacey, 1984). With dropping temperature, *a_w_* value decreased. This trend is consisted with discipline theory (Cao et al., 2018d). This maximum *a_w_* 0.55 indicates food safety which is suggested to be 0.6 for vegetables. Low *a_w_* value is due to loss of free water in the sample at higher temperature. In Figure 5, from 20 to 30°C, *a_w_* value in non‐FDUMC samples was the lowest value while aw value in non‐ADUMC sample was the highest. This result means that FDUMC enables increasing aw value and ADUMC allows deceasing *a_w_* value. At 15°C, FDUMC and ADUMC demonstrated the same law, it was to say that two processing methods decease the *a_w_* value. These phenomena were ascribed to different water adsorption capacities of grass power in different temperatures.

### 3.8 Microstructure

**Figure 6:**
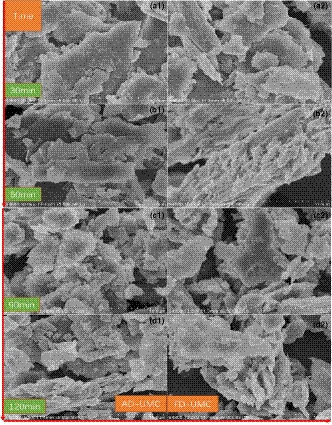
Morphological characteristics (SEM) of barley grass powder using hot air drying/freeze dying combined ultra‐micro‐crushing at different temperatures. *Note:* ADUMC presents air drying combined ultra‐micro‐crushing; FDUMC presents freeze drying combined ultra‐micro‐crushing; a1, b1, c1, and d1 represent barley grass powder SEM with ADUMC at 30, 60, 90, 120 min; a2, b2, c2, and d2 represent barley grass powder SEM with FDUMC at 30, 60, 90, 120 min

Figure 6 shows morphological characteristics of barley grass powder using different UMC profiles at different temperatures. After barley grass processing, these figures demonstrate mostly lamellar morphology and some fewer fragmented pieces. These lamellar structures formed layered structures on fiber bundle (Figure 6‐b2), featuring different thickness and geometry. This phenomenon is ascribed to leafy dried material, which means lamellar structures come from fragments of leafy material. More lamellar structure is also found in other dried vegetables (Akin et al., 1995). With increasing time of processing, fragmented pieces increased and fewer large pieces were produced as shown in illustration. These results were in accordance with particles' diameter change shown in Table 2. From 90 to 120 min, the uniformity of particles was improved markedly, comparing with particles processed for 30‐ or 60‐min UMC. These findings suggested that 90‐ to 120‐min processing time is suitable for barley grass powder. From Figure 6, the subtle difference found between the two processing methods is that ADUMC resulted more debris. The cause might be flexibility of air‐dried material was reduced compared with freeze‐dried one, that is to say air‐dried material possessing methods possess more brittleness. The cause could be higher temperature from air drying impaired fiber bundles and cellulose molecules in which temperature brought brittleness in materials (Hirano et al., 1981). However, morphological characteristics of barley grass powder in ADUMC were high similar to that in FDUMC in 30 min. The results might be due to UMC in 30 min generated the same morphological characteristics because of less importance of two dryings for barley grass.

### 3.9 Sensory acceptability

Table 4 presents the sensory acceptability results of barley grass powder processed by different UMC combined with air freeze drying. Overall comprehensive value was increased with the rise of crushing time. Over 60‐min UMC achieved higher value of evaluation, which implied better acceptance for the experimental group. The reason might be that small particle led better color and appearance (Cao, Islam, Xu, et al., 2020; Cao et al., 2019). The average value and respective value of all samples were above 1.0, which meant all samples were accepted by consumer. This sensory evaluation confirmed the both drying methods followed by UMC provide better product quality of barley grass powder.

## 4 CONCLUSION

In this study, ultra‐micro‐crushing decreases the powder particle size, which was finer in air‐dried than in freeze drying. Ultra‐micro‐crushing is found to increase the total flavonoid and chlorophyll contents of the powders, whereas the WHC and OHC decrease. Ultra‐micro‐crushing is also found to represent the similar behavior in processing odor in freezing‐dried or air‐dried barley grass. 90–120 min is recommended in UMC processing of barley grass powder.

## CONFLICT OF INTEREST

The author(s) declared no potential conflicts of interest with the research, authorship, and publication of this article.

## ETHICAL APPROVAL

This study does not involve any human or animal testing.

## Data Availability

Research data are not shared.

## References

[fsn32138-bib-0001] Akbas, E. , Kilercioglu, M. , Onder, O. N. , Koker, A. , Soyler, B. , & Oztop, M. H. (2017). Wheatgrass juice to wheat grass powder: Encapsulation, physical and chemical characterization. Journal of Functional Foods, 28, 19–27. 10.1016/j.jff.2016.11.010

[fsn32138-bib-0002] Akin, D. , Rigsby, L. , Sethuraman, A. , Morrison, W. , Gamble, G. , & Eriksson, K. (1995). Alterations in structure, chemistry, and biodegradability of grass lignocellulose treated with the white rot fungi *Ceriporiopsis subvermispora* and *Cyathus stercoreus* . Applied and Environmental Microbiology, 61(4), 1591–1598. 10.1128/AEM.61.4.1591-1598.1995 7747973 PMC167414

[fsn32138-bib-0003] Alsuhaibani, A. M. (2015). Biochemical and biological study of biscuit fortified with apple powder. Middle East Journal of Agriculture Research, 4(04), 984–990.

[fsn32138-bib-0004] Cai, Q. , Fan, Z. , Chen, J. , Guo, W. , Ma, F. , Sun, S. , Hu, L. , & Zhou, Q. (2018). Dissolving process of bamboo powder analyzed by FT‐IR spectroscopy. Journal of Molecular Structure, 1171, 639–643. 10.1016/j.molstruc.2018.06.066

[fsn32138-bib-0005] Cao, X. , Islam, M. N. , Duan, Z. , Pan, X. , Xu, W. , Wei, X. , & Zhong, S. (2020). Chlorogenic acid osmosis of snakehead fish: A novel approach to maintain quality and suppress deterioration during storage. International Journal of Food Properties, 23(1), 387–399. 10.1080/10942912.2020.1732409

[fsn32138-bib-0006] Cao, X. , Islam, M. N. , Xu, W. , Chen, J. , Chitrakar, B. , Jia, X. , Liu, X. , & Zhong, S. (2020). Energy consumption, colour, texture, antioxidants, odours, and taste qualities of litchi fruit dried by intermittent ohmic heating. Foods, 9(4), 425. 10.3390/foods9040425 PMC7230953 32260168

[fsn32138-bib-0007] Cao, X. , Zhang, M. , Chitrakar, B. , Mujumdar, A. S. , Zhong, Q. , Wang, Z. , & Wang, L. (2019). Radiofrequency heating for powder pasteurization of barley grass: Antioxidant substances, sensory quality, microbial load and energy consumption. Journal of the Science of Food and Agriculture, 99(9), 4460–4467. 10.1002/jsfa.9683 30868590

[fsn32138-bib-0008] Cao, X. , Zhang, M. , Mujumdar, A. S. , Zhong, Q. , & Wang, Z. (2018a). Effect of microwave freeze drying on quality and energy supply in drying of barley grass. Journal of the Science of Food and Agriculture, 98(4), 1599–1605. 10.1002/jsfa.8634 28833148

[fsn32138-bib-0009] Cao, X. , Zhang, M. , Mujumdar, A. S. , Zhong, Q. , & Wang, Z. (2018b). Effect of nano‐scale powder processing on physicochemical and nutritional properties of barley grass. Powder Technology, 336, 161–167. 10.1016/j.powtec.2018.05.054

[fsn32138-bib-0010] Cao, X. , Zhang, M. , Mujumdar, A. S. , Zhong, Q. , & Wang, Z. (2018c). Effects of ultrasonic pretreatments on quality, energy consumption and sterilization of barley grass in freeze drying. Ultrasonics Sonochemistry, 40, 333–340. 10.1016/j.ultsonch.2017.06.014 28946432

[fsn32138-bib-0011] Cao, X. , Zhang, M. , Mujumdar, A. S. , Zhong, Q. , & Wang, Z. (2018d). Measurement of water mobility and distribution in vacuum microwave‐dried barley grass using Low‐Field‐NMR. Drying Technology, 36(15), 1892–1899. 10.1080/07373937.2018.1449753

[fsn32138-bib-0012] Cao, X. , Zhang, M. , Qian, H. , Mujumdar, A. S. , & Wang, Z. (2017). Physicochemical and nutraceutical properties of barley grass powder microencapsulated by spray drying. Drying Technology, 35(11), 1358–1367. 10.1080/07373937.2017.1332074

[fsn32138-bib-0013] Chantaro, P. , Devahastin, S. , & Chiewchan, N. (2008). Production of antioxidant high dietary fiber powder from carrot peels. LWT‐Food Science and Technology, 41(10), 1987–1994.

[fsn32138-bib-0014] Chouhan, S. , & Mogra, R. (2014). Development and quality evalution of wheatgrass powder. Food Science Research Journal, 5(1), 26–29.

[fsn32138-bib-0015] Coumans, W. J. , Kerkhof, P. J. , & Bruin, S. (1994). Theoretical and practical aspects of aroma retention in spray drying and freeze drying. Drying Technology, 12(1–2), 99–149.

[fsn32138-bib-0016] Frost, S. , Holm, G. , & Asker, S. (1975). Flavonoid patterns and the phytogeny of barley. Hereditas, 79(1), 133–142. 1165204 10.1111/j.1601-5223.1975.tb01469.x

[fsn32138-bib-0017] Havlíková, L. , Šatínský, D. , Opletal, L. , & Solich, P. (2014). A fast determination of chlorophylls in barley grass juice powder using HPLC fused‐core column technology and HPTLC. Food Analytical Methods, 7(3), 629–635.

[fsn32138-bib-0018] Hirano, S. , Tobetto, K. , & Noishiki, Y. (1981). SEM ultrastructure studies of N‐acyl‐and N‐benzylidene‐chitosan and chitosan membranes. Journal of Biomedical Materials Research, 15(6), 903–911. 7309771 10.1002/jbm.820150614

[fsn32138-bib-0019] Huang, L. , Weng, X. , Chen, Z. , Megharaj, M. , & Naidu, R. (2014). Synthesis of iron‐based nanoparticles using oolong tea extract for the degradation of malachite green. Spectrochimica Acta Part A: Molecular and Biomolecular Spectroscopy, 117, 801–804. 10.1016/j.saa.2013.09.054 24094918

[fsn32138-bib-0020] Islam, M. N. , Zhang, M. , Adhikari, B. , Xinfeng, C. , & Xu, B.‐G. (2014). The effect of ultrasound‐assisted immersion freezing on selected physicochemical properties of mushrooms. International Journal of Refrigeration, 42, 121–133. 10.1016/j.ijrefrig.2014.02.012

[fsn32138-bib-0021] Jianrong, H. , Lin, L. , & Bing, L. (2007). The effect of ultra‐fine pulverizing on the quality of food products. Cereal and Feed Industry, 7, 25–27.

[fsn32138-bib-0022] Kamiyama, M. , & Shibamoto, T. (2012). Flavonoids with potent antioxidant activity found in young green barley leaves. Journal of Agricultural and Food Chemistry, 60(25), 6260–6267. 10.1021/jf301700j 22681491

[fsn32138-bib-0023] Karásková, P. , Fuentes, A. , Fernández‐Segovia, I. , Alcañiz, M. , Masot, R. , & Barat, J. M. (2011). Development of a low‐cost non‐destructive system for measuring moisture and salt content in smoked fish products. Procedia Food Science, 1, 1195–1201. 10.1016/j.profoo.2011.09.178

[fsn32138-bib-0024] Magan, N. , & Lacey, J. (1984). Effect of water activity, temperature and substrate on interactions between field and storage fungi. Transactions of the British Mycological Society, 82(1), 83–93. 10.1016/S0007-1536(84)80214-4

[fsn32138-bib-0025] Mujoriya, R. , & Bodla, R. B. (2011). A study on wheat grass and its nutritional value. Food Science and Quality Management, 2, 1–8.

[fsn32138-bib-0026] Nagata, M. , & Yamashita, I. (1992). Simple method for simultaneous determination of chlorophyll and carotenoids in tomato fruit. Nippon Shokuhin Kogyo Gakkaishi, 39(10), 925–928. 10.3136/nskkk1962.39.925

[fsn32138-bib-0027] Peñarrieta, J. M. , Alvarado, J. A. , Bergenståhl, B. , & Åkesson, B. (2007). Spectrophotometric methods for the measurement of total phenolic compounds and total flavonoids in foods. Revista Boliviana de Química, 24(1), 5–9.

[fsn32138-bib-0028] Rana, S. , Kamboj, J. K. , & Gandhi, V. (2011). Living life the natural way–Wheatgrass and Health. Functional Foods in Health and Disease, 1(11), 444–456. 10.31989/ffhd.v1i11.112

[fsn32138-bib-0029] Shu, X. , Wu, Y. C. , Cheng, J. G. , Xia, Y. H. , & Zheng, Y. C. (2007). Mastersizer 2000 laser particle size analyzer and its applications. Journal of Hefei University of Technology (Natural Science), 30(2), 164–167.

[fsn32138-bib-0030] Wang, Q. , Xiong, Z. , Li, G. , Zhao, X. , Wu, H. , & Ren, Y. (2016). Tomato peel powder as fat replacement in low‐fat sausages: Formulations with mechanically crushed powder exhibit higher stability than those with airflow ultra‐micro crushed powder. European Journal of Lipid Science and Technology, 118(2), 175–184. 10.1002/ejlt.201400579

[fsn32138-bib-0031] Yanli, D. (2008). Effect of ultramicro‐pulverization on the astraglus polysaccharides dissolubility. Journal of Anhui Agricultural Sciences, 36(9), 3742.

[fsn32138-bib-0032] YaSha, L. , & Xiaohua, Z. (2004). Current developing status of ultra‐micro grinding equipment. Mining and Processing Equipment, 32(10), 21–26. http://en.cnki.com.cn/Article_en/CJFDTOTAL‐KSJX200410012.htm.

[fsn32138-bib-0033] Zeng, Y. , Pu, X. , Yang, J. , Du, J. , Yang, X. , Li, X. , & Yang, T. (2018). Preventive and therapeutic role of functional ingredients of barley grass for chronic diseases in human beings. Oxidative Medicine and Cellular Longevity, 2018, 1–15. 10.1155/2018/3232080 PMC5904770 29849880

